# Pathways and barriers to tuberculosis care for refugees in Taraba State, Nigeria

**DOI:** 10.3389/fpubh.2026.1828161

**Published:** 2026-10-02

**Authors:** Anna Versfeld, Suraj Abdulkarim, Stephen John, Abdulmujib Abdulkarim, Caoimhe Smyth

**Affiliations:** 1 Department of Sociology and Anthropology, Nelson Mandela University, Gqeberha, South Africa; 2 Stop TB Partnership, Geneva, Switzerland; 3 Sufabel Community Development Initiative, Gombe, Nigeria; 4 Janna Health Foundation, Yola, Nigeria; 5 Independent Consultant, Gombe, Nigeria

**Keywords:** barriers to care, care seeking, health systems, refugees, stigma, tuberculosis

## Abstract

**Introduction:**

Refugee and displaced populations experience high tuberculosis (TB) burdens and face substantial barriers to accessing diagnosis and treatment. Taraba State in north-eastern Nigeria hosts approximately 80,000 refugees, primarily from Cameroon, many living in remote areas with limited TB services. Evidence on how refugees access TB care in this setting remains limited. This study examined the pathways refugees follow from symptom onset to TB diagnosis and identified barriers to accessing and sustaining care.

**Methods:**

A mixed-methods study was conducted between May 2022 and December 2023 in Sardauna, Gashaka, and Kurmi Local Government Areas of Taraba State. Qualitative data included semi-structured interviews with 41 people receiving TB treatment, eight family members, eight community and religious leaders, and eight facilitated discussions with healthcare workers and people affected by TB. Quantitative data were collected through a 15-item Likert-scale questionnaire administered to 130 people receiving TB treatment. Qualitative data were thematically analyzed, while quantitative data were analyzed descriptively.

**Results:**

Refugees described prolonged and complex pathways to TB diagnosis. Symptoms were often initially attributed to common illnesses, leading most participants to first seek care from patent medicine vendors or traditional providers. Misdiagnoses across sectors contributed to substantial delays, with a median time to diagnosis of 7 months and longer delays among women. Participants reported high out-of-pocket expenditures before diagnosis. Our sample focused on people who received a TB diagnosis. The tenacity, resources, and resilience for diagnosis they described suggest that many others likely went undiagnosed. Major barriers to accessing and sustaining care included food insecurity, transport and healthcare costs, anticipated stigma, language barriers, long distances to facilities, poor facility conditions, and negative experiences with healthcare providers.

**Discussion:**

Refugees in Taraba State navigate long, costly, and often arduous journeys before receiving a TB diagnosis, frequently consulting multiple providers and exhausting scarce resources in the process. Multiple, gendered barriers to care make continued care access an ongoing challenge. These findings underscore both the persistence of people seeking care and the systemic barriers that shape access to TB services. Without addressing these structural constraints, earlier diagnosis and equitable TB care for displaced populations will remain difficult to achieve.

## Introduction

Tuberculosis (TB) remains a major global public health challenge, with displaced and refugee populations experiencing particularly high TB burdens (1) and significant barriers to accessing care. These barriers affect not only the individuals and families affected directly but also broader efforts to reduce TB transmission and improve population-level TB outcomes. Therefore, providing effective TB care for refugee populations is an urgent global challenge (2).

Taraba State in north-eastern Nigeria has an estimated population of approximately 3.2 million people and has experienced substantial population displacement due to years of political instability and conflict in the surrounding region. In addition to a large number of internally displaced people in the area, approximately 80,000 refugees—primarily from Cameroon—are settled on the Mambilla Plateau and within host communities across Sardauna, Gashaka, and Kurmi Local Government Areas, including the remote Ngada Special Development Area (SDA). In 2022, when this research was initiated, 206 health facilities served these areas. Of these, 107 provided Directly Observed Treatment Short-course (DOTS) services, while only 16 offered TB diagnostic services, supported by a single GeneXpert machine.

Refugees are a noted vulnerable population in Nigeria, and access to healthcare in these areas remains limited. Refugees and host communities frequently rely on traditional health practitioners and patent medical vendors (PMVs)—local, informal pharmacy shops.^1^ Barriers to care include the centralisation of TB services, limited human and financial resources, and service delivery models that are not tailored to the needs of displaced populations. In addition to federal and state government programs, non-governmental organizations play an important role in providing TB services for refugees and internally displaced populations.

Literature on internally displaced populations in the region describes the multiple health challenges faced, including high rates TB and HIV (3), acute malnutrition, and maternal morbidity and mortality (3). Noted TB care access challenges include distance to care facilities, costs of transport and care, and waiting times (4). Studies from other contexts emphasize the importance of designing services that respond to the specific needs of refugee populations being served, such as decentralized care that is available free of cost in refugee languages (5, 6). However, there is little local evidence describing access to TB care among refugee populations, who face additional challenges related to their legal and social status (2). Understanding how refugee populations experience the pathway from symptom onset to TB diagnosis and treatment is critical for designing services that can effectively reach and retain these populations in care.

Supported by the Stop TB Partnership Challenge Facility for Civil Society (CFCS), which strengthens civil society organizations to expand access, improve care quality, and promote sustainable TB services, this study examined refugees’ experiences accessing TB care in Taraba State. Specifically, the study sought to document the pathway from the first onset of TB symptoms to diagnosis and to identify perceived barriers to care among refugees currently receiving TB treatment. By examining these experiences, the study seeks to inform approaches that can improve access to TB services for displaced populations in this, and other, similar contexts.

## Methods and approach

This mixed-methods study examined barriers to TB care among refugee populations in Taraba State. Qualitative data included eight semi-structured interviews with community and religious leaders (all men) to explore local health contexts and challenges. Eight facilitated discussions with healthcare workers and people affected by TB combined focus group discussions and prioritization exercises to identify key barriers and facilitators. Forty semi-structured interviews with people affected by TB (18 men and 22 women) examined care-seeking practices, costs, and pathways, including development of care access timelines. Eight interviews with family members accompanying TB patients (five men and three women) captured perspectives of close contacts supporting care.

Participants for all qualitative components were purposively sampled through public TB facilities (though some had previously accessed private facilities—see “Pathways into care” below). Invitations extended to individuals meeting study criteria on the days researchers were present. Early qualitative analysis informed a 15-item Likert-scale questionnaire capturing perceived barriers to care. The questionnaire was interviewer-administered to 130 people with TB currently in treatment using a 5-point scale from “strongly agree” to “strongly disagree.”

Data collection occurred between May 2022 and December 2023 and was conducted by three refugees trained as researchers who were recruited from the study communities, minimizing language barriers and supporting trust. Interviews were conducted in English, Hausa, or Fulfulde, transcribed and translated by the team, and checked by the research lead.

Qualitative data were thematically analyzed using NVivo 12, first inductively to identify emergent themes around symptom recognition and care-seeking and then deductively in relation to questionnaire items. Coding was conducted by one main researcher and validated through team discussion. Quantitative data were analyzed in SPSS and cross-checked in Excel. Interview guides were iteratively adapted to explore emerging issues. Written informed consent was obtained from all participants; no compensation was provided.

## Findings

### Pathways to into care

This section presents findings on pathways into care for people with TB. Data come from semi-structured interviews, including timeline interviews and expenditure estimates. Overall, 41 (19 men and 22 women) people affected by TB, aged 19–60 years, were interviewed (Figure 1). Where possible, counts of particular responses have been tallied and related to the total number of participants answering each question.

**Figure 1:**
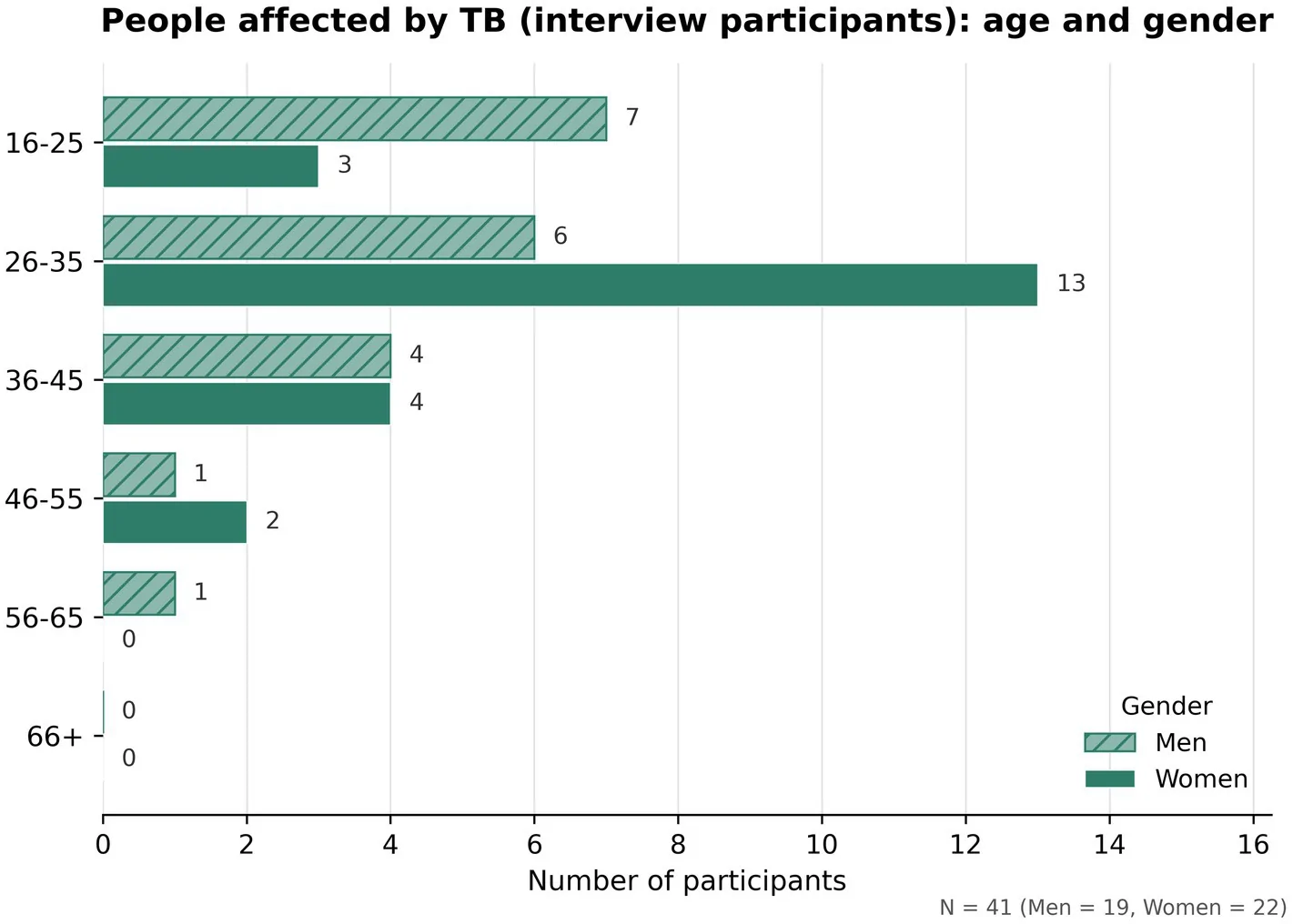
Interviewee demographic data.

The findings are organized into five areas. “Recognising TB disease” examines how people interpreted early TB symptoms, including cough, weakness, fever, and loss of appetite, and how these were initially misattributed to common illnesses or seasonal changes. “Health-seeking” describes how and where care was sought, outlining the range of providers consulted—PMVs, traditional healers, private clinics, and public facilities—and patterns of repeated visits, reflecting both perceived severity and available resources. “Care responses” details (mis)diagnoses and related treatments received from providers. “Time to diagnosis” reports the interval between symptom onset and TB diagnosis, while “Costs of care seeking” estimates financial expenditure during this period. Collectively, these themes illustrate the extreme tenacity required of refugees with TB to obtain a diagnosis.

### Recognizing TB disease

Men and women affected did not readily recognize that they had tuberculosis from the symptoms, and delays to health seeking were often related to misinterpreting disease cause, or minimizing symptoms as not being serious. Initially, tuberculosis disease was experienced as a range of symptoms including weakness (often noted as an inability to work or walk uphill) and a cough—frequently described as transforming from a “regular” cough to a whooping cough. Four participants indicated coughing blood as a symptom—suggesting advanced illness before treatment initiation (see time to diagnosis, below).

A wide range of other symptoms were also described, including catarrh (excess mucous), loss of appetite, headache, fever, shivering, night sweats, body aches, dry or itchy throat, heartburn, stomach pain, chest pain or feeling of a “blockage” in the chest, and vomiting. In the absence of knowing about TB, symptoms were initially mostly interpreted as resulting from common illnesses (flu, malaria, or typhoid). Often, the start was linked to seasonal change, or the rainy season and exposure to weather, especially through working outside. Perceptions that symptoms were not serious were described as changing with time, as they worsened or—though not lessening—became regarded as “stubborn” and therefore in need of treatment.

“The first time my sickness started was just like a joke, it began with common ailment that I never thought was very serious, just fever! I was experiencing fever with a strong headache (a very severe one) disturbing me, I took all the necessary medications that could make me well over long time of treatment but all in vain.” (Interview, person affected, man 20 years).

“The first time I notice this was few months ago during the cold weather of the rains when heavy rain beat me on my way home, the weather was so cold that I was shivering all night long, and I wake up with catarrh.” (Interview, person affected, woman, 20 years).

### Health-seeking

Participants sought care for symptoms of poor health from a range of providers, including PMVs, traditional healthcare providers, private facilities (such as doctors, clinics, and hospitals), and the public sector (clinics and hospitals). The majority (62%, *N* = 25) first sought care from PMVs, making it the most common initial point of care.

The public sector was the next most frequently accessed, although only 15% (*N* = 6) of participants seeking care there first. Traditional healthcare providers were the first choice for 10% (*N* = 4), while 7.5% initially sought care from private facilities.

Seeking care at pharmacies was often linked to an initial perception that symptoms were not serious. When treatment from one pharmacy did not lead to a cure, participants often returned to the same pharmacy multiple times or tried different medications from various pharmacies. While some participants noted that pharmacy treatments provided symptomatic relief, others viewed the failure of these medications as an indication of a more serious illness and need to seek care elsewhere.

All but one participant sought care from multiple providers rather than relying on a single source. All except one reported accessing care from more than one location, either by visiting multiple providers within the same sector (e.g., different pharmacies or traditional healers) or by engaging with different types of service providers (e.g., both pharmacies and traditional healthcare providers).

Although less commonly the first point of care compared to PMVs, traditional healthcare providers were widely used at some stage of the care-seeking journey, often consulted alongside other health-seeking efforts or when other treatments had been unsuccessful. The majority of participants indicated that they had turned to traditional healthcare at least once.

“I took some medications from a nearby [patent medical vendor] without consulting any provider thinking it was just a normal malaria and catarrh.” (Interview, person affected, man, 45 years).

“I tried self-medication at first, having simple tabs from medicine vendors around my area, when it did not work for me I tried traditional medication for months but no much progress, I went to different health care centres and tried different treatments, it did not work.” (Interview, person affected, woman, 29 years).

“The traditional healer I visited before wasn’t a strong one in his line of work, but I met a stronger one in Batcha, that proudly said he could cure me, but…still the same no cure after I gave him almost four chickens, though the illness subsided for some time.” (Interview, person affected, man 40 years).

### Care responses

Across all sectors where healthcare was sought, misdiagnoses were common, with participants reporting diagnoses of flu, malaria, typhoid, yellow fever, ulcers, and allergies, among other conditions. Pharmacies frequently provided treatments that offered temporary symptom relief, with care providers adapting the medication provided to fit the budget of the person affected. Traditional healthcare providers primarily offered herbal remedies, including purgatives, which, such as pharmacy treatments, often provided temporary relief but delayed further care-seeking. When one traditional healer’s treatments were ineffective, participants commonly sought care from another, mirroring patterns observed with pharmacy visits.

Private sector care was widely described as leading to misdiagnoses and expensive treatments, while public clinic attendance also resulted in misdiagnoses. Of the four participants (10%) who first accessed public clinics, none were diagnosed at that stage, and of the two who sought care at public hospitals, only one received a TB diagnosis on their first visit. (This individual had immediately accessed a public hospital in December 2020 due to fear of COVID-19.) Regardless of where they sought care, all participants who accessed health facilities received some form of medication. Almost a third (9/30) were placed on an intravenous drip at least once, and nearly a quarter (7/30) reported receiving at least one injection. Some participants described receiving repeated injections—each of which required payment—including one who reported receiving nine injections and another who received “about 20.”

“I felt fever and I went to health clinic to see a doctor, and they checked blood pressure and told me that I was having yellow fever. I took 9 injections in just 3 days, and still no cure and my body started becoming weak, the day I finished taking the injections, My body continued to get weaker and weak.” (Interview, person affected, woman, 33 years).

“I went to a [patent medical vendor] bought medicine and took it for few days and it relieved me (partially) for some days. But even with that when I cough I will be having (mucus), I still thought it was ordinary cold (flu).” (Interview, person affected, woman, 33 years).

### Time to TB diagnosis

Extended periods of seeking care from multiple places before TB diagnosis resulted in delayed diagnosis for the majority of research participants, with the most extreme being a reported 11-year gap between first seeking care for symptoms and TB diagnosis (woman, 29 years). Of the 26 participants reporting on the time between symptom onset and diagnosis, only one person reported immediate diagnosis. Just under a quarter (23%, *N* = 6) were diagnosed in 3 months or less, and for over half the participants, it took 6 months or more to get a diagnosis after symptom onset. It took women considerably longer to get diagnosed than men, with the median time to diagnosis for women (*N* = 14) being 9 months, and the median time to diagnosis for men (*N* = 12) being 5 months. For all participants, once they provided sputum for TB testing, diagnosis was within a few days, and for most within the same day.

### Cost of care-seeking

Estimating the cost of health seeking was difficult for many participants. For some, this was because the interviewee had not managed the costs themselves, others had simply lost count. However, 23 participants (12 women and 11 men) reported that they were able to calculate or estimate the costs of seeking treatment up until the time of diagnosis. These costs ranged from a minimum of N5,000 to a maximum of N270,000 (approximately USD 12 USD 648, respectively).^2^ The mean cost was N101,000 (USD 242.4), and the median cost was N100,000 (USD 240). These high costs were explained in terms of the extended time getting a diagnosis had taken, the range of providers visited and treatments provided, and the additional related costs, such as transport.

“…even before I started the traditional treatment I spent money in the chemist about N5,000 within the first month, then the traditional medicine I spent more than N30,000 all in vain as it did not cure me, nothing changed. When I went to the hospital I spend almost N100,000 for earlier treatment of the fever, flu and other sicknesses which all went for nothing within that three months. Then I went back to the hospital again I spend N6000 for different tests and treatment.” (Interview, person affected, woman, 32 years).

### Barriers to continued care access

The findings from the Likert-scale questionnaires (reported as percentages) are presented alongside qualitative data from interviews and focus group discussions. The results are organized into three categories—individual, interpersonal, and health system factors—drawing on a meta-synthesis of barriers to TB care in Nigeria (7).

Individual barriers include personal circumstances such as hunger, inability to meet needs beyond basic survival, costs of transport and services, food while attending care, time away from income-generating activities, and access to socially appropriate clothing.

Interpersonal barriers encompass social dynamics, including language, stigma, self-efficacy, and healthcare provider attitudes, which reflect broader social positioning and refugee status.

Health system barriers cover factors relating to care provision, including facility location, infrastructure, medication availability, and perceived quality of care.

These categories are analytic rather than mutually exclusive; barriers often overlap and reinforce each other. For example, distant facilities (a health system barrier) increase demands on personal resources such as time and money. This framework facilitates comparison across findings and highlights how barriers cluster, while also offering new insights on refugee experiences in Nigeria (Figure 2).

**Figure 2:**
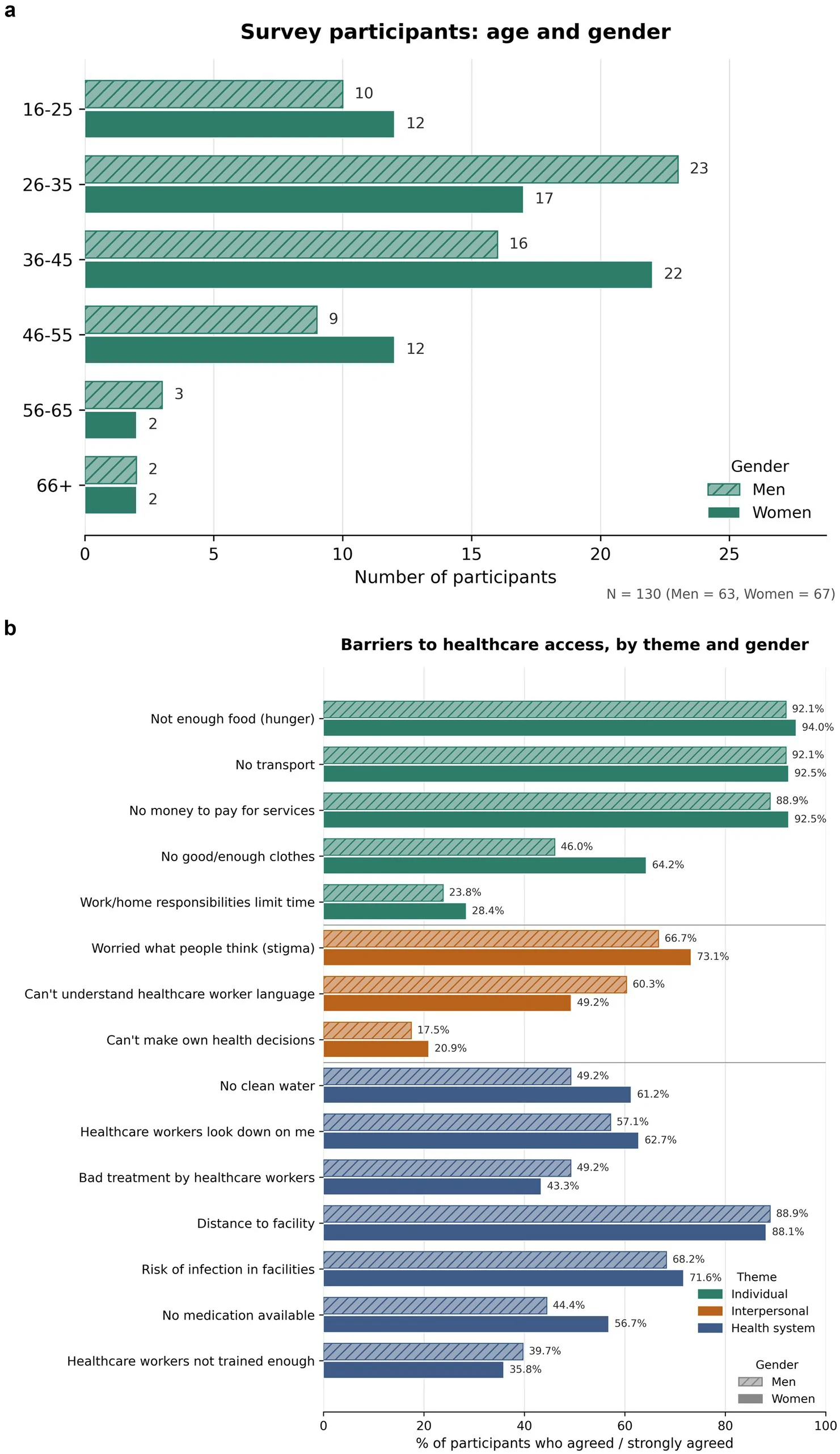
Survey results **(a)** Demographic data **(b)** barriers to care access.

### Individual barriers

#### Hunger

The vast majority of participants (93.08%) agreed or strongly agreed that not having enough food was a barrier to accessing care. This was marginally higher (94.03%) in women than in men (92.06%) but was the most widely stated barriers for all genders. Participants explained that conditions of poverty and related hunger were key challenges faced in day-to-day living and that hunger deprioritized care access. Insufficient access to food was as a key barrier to taking treatment, especially when healthcare providers emphasized the need for food.

“The doctor will ask you have you eaten? If you do not, he will tell you to go and eat and sometimes you do not have money to buy the food or there’s no food available to buy in the hospital premises. Some drugs you cannot take them without eating food because they are very strong.” (FGD, People affected by TB).

#### Work and other competing priorities

Overall, the generalized conditions of poverty were the most frequently described as an overarching challenge for care access described in the qualitative research, and a quarter (25.38%) of participants indicated that immediate home and work requirements served as a barrier to care. This was somewhat higher for women (28.36%) than it was for men (23.81%). The qualitative research suggests that for women this was related to care requirements within the home, and for men it was related to farming or other income generating work.

“Our life is reduced to only struggles for what to eat and feed our family, and we only think of going to health facility when we have to or when it is absolutely necessary. Most of us will be managing their sicknesses for days but we do not see going to the facility as an option until when one’s condition is critical…” (Focus group discussion, people affected by TB).

“We do not have means to cultivate our farms which is our only means of providing for our families…So visiting health facility is only when we are taken there in critical condition.” (Focus group discussion, people affected by TB).

#### Transport costs

In total, 92.31% of participants agreed that transport costs were a barrier to care, making this the second most noted care access barrier. Respondents described having to save money for the journey to the hospital, only to find themselves unable to afford hospital bills or the return trip. One participant noted that if the hospital was closer, they could visit to request support even without resources for treatment, highlighting transport costs and distance as primary barriers to care. Additionally, participants mentioned that after making the costly journey, they were sometimes sent home and told to return the following day.

“[By the time] you get money your sickness is at [its worst]. And when you get to the hospital, you have no money with you for other hospital bills. And you cannot walk your way from home to the hospital because is very far.” (Focus group discussion, people affected by TB).

#### Absence of personal resources for service costs

The vast majority of participants (90.77%) agreed or strongly agreed with the statement, “I do not have money to pay for services.” This concern was more prevalent among women (92.54%) than men (88.89%). Notably, all participants aged 56 and older indicated that this was a concern for them.

In qualitative discussions, participants noted the competing costs of transport and medical care, stating that they could afford one or the other, but not both. Visiting the hospital was also associated with additional expenses, such as food and water. Beyond being a practical barrier, the inability to pay was described as an emotional one as well, navigating the healthcare system without sufficient funds was seen as a humiliating experience.

“If you do not have a money that can satisfy your daily needs you cannot afford to take care for TB or any other disease.” (Focus group discussion, religious and community leaders).

### Interpersonal barriers

#### Stigma and discrimination

The majority (70%) of participants said that being worried about what other people would think about them was a care access barrier. This was more for women (73.13%) than for men (66.67%). Shame and self-stigma were described as being compounded by the double burden of having TB and being a refugee. Community leaders highlighted that fears of stigma and discrimination may be worse for women, who may fear being marked as unmarriageable, or as having HIV.

“We feel embarrassed in the facility due to our illness, and so most of us prefer to stay at home with our condition and seek for traditional solutions than to visit the facility. This is in addition to the normal stigma we are experiencing from other people in the community as people are afraid of being infected with diseases….” (Focus group discussion, people affected).

“[Women] also think that if they are diagnosed with TB they’ll not get married and no one will ask their hand in marriage, but men do not have that kind of fear.” (Focus group discussion, religious and community leaders).

#### Lack of clothing deemed respectable

Over half (55.8%) of participants either agreed or strongly agreed with the statement, “I do not have good/enough clothing.” This was notably higher in women (64.38%) than in men (46.03%). Acquiring new clothing was not a priority in the context of extreme poverty and that this, in turn, was cause for shame and feelings of inferiority, restricting movement and health facility access. Not having decent clothing was further seen to be a problem because healthcare workers were described as assessing the value of patients through their appearance—providing less, or poorer quality care, to those wearing clothing which marked them as living in poverty.

“In the condition of hunger, clothing does not become a priority even as it is necessary, for most of us who could not afford buying the cheaper ones we prefer to stay where we will not be seen at all than going to the health facility where one will be looked down upon due to the garments he puts on even with the sickness.” (Focus group discussion, people affected).

“[Healthcare workers”…try to rate your status before they interact with you in the health facility. If they see you coming to the facility with car or wearing good dresses they will honour you, [treating you with] you regard.” (Focus group discussion, people affected).

#### Self-efficacy

Inability to make personal decisions about health was described as a barrier by 17.69% of participants. This was slightly higher for women (20.90%) than it was for men (17.46%). Notably, in the age group 56–65, the majority (60%) indicated they were not able to make their own health decisions, suggesting self-efficacy lessens with age.

Restrictions on women’s decision-making and freedom of movement were identified as key barriers in focus group discussions. Participants noted that, within the religious and cultural context, women often required permission from their husbands to make decisions, take action, or leave the home, particularly when financially dependent on men. Additionally, religious and cultural norms restricting women from receiving healthcare services from man providers were described as limiting access to care in settings where woman healthcare providers were unavailable.

“one of the reasons is economic factor in such a way that women in our community do not have freedom to work and live freely on their own, they either depend on their parent or spouses, because of that they are marginalized and all their hopes are on men to provide for them.” (Focus group discussion, religious and community leaders).

“In some religions women are not permitted to associate with men, some men also prevent their wives from going to receive treatment [from man providers]…” (Focus group discussion, religious and community leaders).

#### Language

Just over half (54.6%) of the questionnaire participants indicated that language barriers with healthcare providers were a concern for them. This was greater for men (60.32%) than for women (49.25%). Refugees mostly spoke English, while host communities and service providers use Hausa or Fulani—making it challenging to communicate about illness experienced.

#### Health system barriers: distance, infrastructure, and care quality

A range of barriers related to the quality and responsiveness of service provision were noted. These included availability challenges, which translate to challenges related to distances that need to be traveled to access care, facility infrastructure, and conditions.

#### Traveling distance and road infrastructure challenges

The dispersed nature of healthcare facilities and the distances people had to travel to access care were a considerable challenge, compounding the difficulties of covering transport costs. A total of 88.46% of respondents agreed or strongly agreed with the statement, “The journey there and back is too far.” This perception varied little between men and women. Distance was cited as a barrier to care due to poor road conditions and difficulties in finding transport.

Traveling long distances were also described as physically demanding, particularly for those who were unwell. Poor road conditions and the physical toll of traveling were described as an additional health risk for sick individuals. While most negative health impacts were linked to worsening illnesses due to difficult travel conditions, some also pointed out additional risks during the rainy season, such as overflowing rivers and slippery, muddy roads that made travel even more hazardous.

“The road itself is not motorable and during rainy season it is even worst, is not everybody that will agree to assist patient through the health facility road. So after distance we have to think of the terrain too, if not out of necessity one will not like to follow.” (Focus group discussion, person affected).

“If you take your patient be it inside a car or on a bike because of how bad the road is and so many potholes your patient will get extremely tired from the journey you might end up taking a dead body to hospital because your patient died along the way.” (Focus group discussion, person affected).

#### Poor facility infrastructure and facility conditions

Participants in the qualitative research noted that previous experiences of poor infrastructure and unhygienic conditions influenced willingness to return to healthcare facilities. Concerns about hygiene conditions in the facilities and nosocomial disease spread were captured in the questionnaire through responses to the statement “*I am worried I will catch another disease*” [at the facility]. Over 70% of participants (71.64% women and 68.25% men) agreed that this concern served as a barrier to care access. Limited access to potable drinking water in healthcare facilities was also noted as a barrier to care for 55.38% of participants. This issue was reported more frequently by women (61.19%) than by men (49.21%).

Qualitative research participants noted that non-potable drinking water was a major factor contributing to poor health within the research communities and that buying bottled or sachet water was only an option for those with sufficient resources. At home, drinking water could be boiled to improve its quality; however, this was not possible in health facilities. Water access was particularly concerning during longer facility visits, with respondents expressing fears about using well water and the associated risk of contracting diseases such as typhoid. Lack of water also prevented people from washing themselves, their clothing, or—for in-hospital stays—their bedding. This was described as doubly challenging as it could result in complaints or reprimands from healthcare providers.

“When there’s no windows, doors, ceiling, bad floor, everywhere is open and there’s cold. You left the comfort of your home and come to where you’ll end up getting another disease without treating the one you have.” (Focus group discussion, person affected).

“…you’ll end up with a dirty and smelly room[…]and the doctors will be complaining about it and is not our fault because there’s no water.” (Focus group discussion, person affected).

#### Lack of medication availability

Just over half (50.77%) of participants strongly agreed or agreed with the statement, “The health facility does not have the medications I need.” Qualitative discussions about medication availability included being sent elsewhere to buy medication and being prescribed paracetamol no matter what their health concern (sometimes after being required to undertake a range of assessments).

“And sometimes they’ll say bring money we will buy the drugs for you from outside. If they can buy from outside why will not I? Then there’s no need of coming to the hospital” (Focus group discussion, person affected).

“For instance, you went to hospital and some tests were done and the result came out. Instead of you to be given drugs in the hospital they’ll just write for you and ask you to go to local pharmacy and buy.” (Focus group discussion, person affected).

#### Stigma and discrimination—healthcare providers

Perceived and enacted stigma and discrimination were noted in health facilities. In total, 60% of participants agreed or strongly agreed with the statement, “I feel like the healthcare workers look down on me.” Women expressed somewhat greater concern about healthcare worker attitudes than men, with 62.69% of women agreeing with this statement compared to 57.14% of men. Furthermore, 46.15% of participants also agreed with the statement, “The healthcare workers do not treat me well.” This concern was slightly higher among women (49.21%) than men (43.29%).

Healthcare workers were described as providing care in an untimely, unprofessional, and discourteous manner to individuals perceived as being of lower social status or unlikely to afford services. Reports of poor treatment included being made to wait unnecessarily, not being listened to, or being spoken to in a dismissive or derogatory manner by nurses and doctors.

“…if the workers realised [the patient] does not have money for the services or medications, one hardly gets proper attention and may not be having chance to voice out his problems…” (Focus group discussion, person affected).

“Maybe [healthcare workers] know that nobody is supervising them, or [maybe] nobody cares to know how they treat the poor refugees so they do it the way they want and the patients leave them and prefer to keep distance from them than face embarrassment.” (Focus group discussion, person affected).

#### Insufficient provider expertise

39.23% of questionnaire participants (39.68% of women and 35.82% of men) agreed with the statement, “The Health Care Workers are not trained or knowledgeable enough.” Qualitatively, participants described being prescribed different medications for the same condition by different providers, or being provided incorrect medication. This was described as sometimes resulting healthcare providers lacking in expertise to make the corrected diagnoses or from a lack of care.

“And sometimes you will find unqualified Doctors that do not know their work. If you are having headache, they’ll put drip for you, stomach pain they’ll give you drip. Any small thing is just drip.” (Focus group discussion, person affected).

#### Ancillary costs

The ability to pay for services as a key barrier to care (see above) was exacerbated by a wide range of out-of-pocket medical expenses described by participants. Despite mandated free TB medication, a range of ancillary costs were described including, for example, payments required for opening a folder, for additional laboratory or blood tests, or for treatment of comorbidities.

“Visiting the Health facility involves opening folder which cost money, then if it require visiting lab for scanning or any other test one most pay money there too, so the issue of money is so big that it could not be overemphasized.” (Focus group discussion, person affected).

## Discussion

### Journey to diagnosis

Our study highlights the complex and prolonged paths that refugees navigate before receiving a TB diagnosis. Initial symptom recognition was low: Participants often attributed poor health to seasonal changes, exposure to wet weather from outdoor work, or common acute infections such as malaria, typhoid, or flu, rather than TB. Unlike some previous studies, we did not find attribution to spiritual causes (8, 9). We also found consistent minimisation or misattribution of TB symptoms, likely related to low rates of knowledge about TB (7, 9, 10), but potentially also a result of the fact that physical distress widely experienced as a result of difficult life circumstances. Overall, recognition of serious illness was less related to symptom severity than to symptom duration, suggesting a temporal interpretation of illness that must be taken into account in awareness processes.

Care-seeking pathways were diverse, with most participants initially consulting patent medicine vendors (PMVs), followed by public, traditional, and private providers. This reliance on PMVs has been noted elsewhere (11–14). This implies that a first step in reducing the time to diagnosis must include the engagement of PMVs. PMVs have been found to be receptive to being included in the TB response in Southern Nigeria (15) and contributed to TB case finding during COVID-19 (16). Furthermore, as trusted care providers, PMVs also potentially have a valuable role to play in creating TB awareness in the communities they serve (16).

We also found that while traditional care providers were less frequently the first point of care, almost all participants accessed traditional healthcare providers at least once in their care-seeking journey, implying that this is also an important group to include as partners in tuberculosis screening, education, and linkage to care. A study in Lagos (17) found high levels of willingness in traditional healthcare providers to support TB diagnosis and care. This should be further explored in Taraba State.

Participants did not (except one) receive a TB diagnosis at their healthcare provider first visit, even if these were biomedical care providers, reinforcing concerns about provider awareness and diagnostic capacity across sectors (14, 18). Misdiagnoses—including malaria, typhoid, yellow fever, ulcers, and allergies—further delayed effective care, underscoring the need for enhanced training and incentives for providers, including PMVs, private practitioners, and traditional healers to support timely referral and diagnosis.

Our findings provide new insights into the treatments received as a result of misdiagnoses. Participants reported using purgatives, symptom suppressants, and intravenous drips or injections. These interventions, while sometimes temporarily relieving symptoms, do not treat TB and may carry additional health risks, including potential exposure to HIV or hepatitis C (from IV drops and injections). The provision of antibiotics may contribute to antimicrobial resistance—including the development of drug-resistant tuberculosis—through inappropriate use of medications such as streptomycin, which is widely available in Taraba State. Misguided biomedical treatments may also drive people toward repeated traditional care, further delaying effective TB management. This all points toward the need for comprehensive programs to broaden the range of care providers who are actively seeking TB and bringing people into quality TB care.

The cumulative effect of multiple care-seeking attempts resulted in substantial delays, with a median of 7 months from symptom onset to TB diagnosis—markedly longer than the approximately 2.5-month interval reported elsewhere in Nigeria (19). This likely reflects the precarious conditions and additional barriers to care faced by refugees. Women experienced exacerbated access challenges (7, 14) particularly prolonged delays (12, 20), with a median of 9 months to diagnosis versus 5 months for men, highlighting the intersection of gender, poverty, and refugee status in shaping barriers to timely care. However, given our limited sample size, we suggest that the extent to which these gendered differences are more broadly experienced warrants further exploration.

Delays and repeated attempts to access diagnosis resulted in high costs of care-seeking—approximately N100,000 (USD 145) *before* diagnosis was reached. In a context of widespread poverty and reliance on subsistence farming, where USD 1 bought almost 2 kilograms of maize meal, this likely pushed families further into poverty (11, 21, 22).

Research team members who working in the area also noted that the perceived efficacy of treatment is often related to the cost—this may shape local willingness to pay for treatment, despite hardships, even in the absence of information or evidence about efficacy. This suggests the need to provide additional information highlighting that TB treatment as efficacious even if it is provided free of charge.

Finally, our study captures only those who ultimately reached the public sector and received a TB diagnosis, meaning these experiences represent the “lucky ones”—the people with the financial resources (however, strained) and social support to continue seeking care despite extreme barriers. Others may have faced insurmountable obstacles, remaining undiagnosed and untreated.

Taken together, these findings about complex pathways into care point to the need for a collaborative, cross-sector approach to finding TB that engages the full range of providers people affected actually turn to formal and informal alike. Equipping all of these providers with basic TB knowledge, verbal screening skills, and clear pathways for further diagnosis or referral could help address the access barriers described above. Achieving this will likely require attention to providers’ incentives: Since ongoing provision of medications such as antibiotics generates income, while referral to the public sector may result in a financial loss for traditional or private providers, referral pathways may need to be actively incentivized rather than assumed.

### Barriers to care

#### Individual barriers to care: food insecurity and costs

Food insecurity is common among refugees and internally displaced people in Nigeria (4) and emerged as a critical barrier to care among refugees in our study. Hunger was the most frequently reported individual-level obstacle, cited by 93% of participants. This reflects a harsh reality: Attending a health appointment can mean choosing between seeking care and securing food for oneself or one’s family. Food insecurity not only threatens treatment adherence (23, 24), it can prevent access to care altogether.

Financial constraints compounded these challenges. Transport costs and healthcare fees were reported as barriers by 92.3 and 90.8% of participants, respectively. Despite TB services being officially free, additional expenses, including administration, diagnostics, X-rays, and medications, persist (25) placing significant strain on people and households. Previous studies in Nigeria corroborate these findings: Transport costs account for a substantial proportion of missed appointments (26) and contribute to catastrophic expenditures for TB-affected individuals (21).

These individual-level barriers illustrate the impossible trade-offs refugees with TB must navigate. Stopping work to attend appointments may lead to hunger; paying for transport or healthcare may compromise other essential needs. Such pressures extend beyond physical health, contributing to psychological stress and distress that can persist throughout TB treatment.

While we have framed costs and food insecurity as individual barriers, are not experienced in isolation—they are shaped by broader structural factors, including poverty, geographic marginalization, and the challenges of displacement. Addressing food insecurity, transport, and healthcare costs requires interventions that consider these structural determinants, alongside expanded psychosocial support programs that reach all people with TB, including those with drug-sensitive disease. Decentralized care or mobile services provided closer to where refugees work or live could also go a long way to reducing food, transport, and cost barriers.

#### Interpersonal barriers to care: stigma, dignified dress, and language barriers

TB stigma is widespread in Nigeria, and anticipated stigma was a major barrier to care access, reported by over two-thirds of participants. This aligns with literature documenting TB stigma as a barrier to care which contributes to delayed diagnosis and treatment challenges (7, 27). Fear of stigma may be amplified by high rates of HIV among refugees (3), creating a potential triple stigma burden: refugee status, TB, and HIV. Stigma was more frequently reported by women than men, likely reflecting the harsher social consequences women may face, such as divorce, if diagnosed with TB (7, 28).

Our study also highlighted the relationship between anticipated stigma and dignity, particularly regarding access to clothing suitable for clinic visits. This barrier was more common among women, suggesting either limited access to appropriate clothing or heightened social scrutiny. Addressing this requires practical support—such as providing suitable clothing and footwear—alongside training for healthcare providers to reduce stigma and discrimination.

While restrictions on women’s movement were raised in qualitative interviews and have been noted by others (7, 14, 29), our questionnaire data did not show substantial differences in self-efficacy between men and women. Challenges were more pronounced among older participants, with 60% of those over 55 reporting their inability to make their own health decisions as a care access barrier, highlighting the need to consider older populations in TB programming.

Language barriers were reported by over half of participants, reflecting difficulties communicating with healthcare providers—demonstrating a specific challenge for refugees. Language barriers were reported slightly more by men than women, although the reasons are unclear. Expanding language support services could improve care accessibility and experiences for refugee populations.

#### Health system barriers: geography, health facility quality, and care provider knowledge and attitude

Geographic access remains a major structural barrier to TB care in Taraba State. Participants frequently described the difficulty of reaching health facilities, particularly where poor road infrastructure and seasonal conditions make travel unreliable. Similar challenges have been noted elsewhere in Nigeria, where distance and transport costs significantly affect access to TB services (22). These findings reinforce the importance of decentralized service delivery or targeted support for travel, particularly in rural areas where refugees and host communities often live.

Concerns about the condition of health facilities also shaped perceptions of care. A recent assessment of facilities in Taraba State validated our findings finding widespread infrastructure limitations, including inadequate sanitation, limited water access, and unreliable power supply (30). Such conditions extend beyond questions of comfort; they pose health risks and can undermine trust in health services, especially when people in care are held personally to cleanliness standards that are not possible under the circumstances. Strengthening basic infrastructure and ensuring minimum sanitary standards are therefore essential components of improving TB service accessibility.

Participants also raised concerns about medication availability. While our study did not distinguish whether these concerns referred specifically to TB medicines or to broader health system shortages in the state (30), TB drug stock-outs are a well-documented problem in Nigeria, particularly for drug-resistant TB (14, 31). Perceptions of unreliable medication access may therefore contribute to broader skepticism about the effectiveness of public healthcare.

Experiences within healthcare facilities were another important dimension of access. TB-related stigma among healthcare providers has been widely documented in Nigeria, including behaviors such as avoidance, shaming, or blaming of people affected (7, 27). Our findings suggest that such dynamics may be compounded for refugees, who can experience layered forms of marginalization when engaging with health systems (32, 33). Addressing stigma in TB services therefore requires attention not only to TB-related discrimination within healthcare settings but also to the broader social marginalization that shapes refugees’ expectations and experiences of care.

Finally, participants’ concerns about healthcare provider knowledge point to broader systemic challenges. When considered alongside the repeated missed opportunities for TB diagnosis described earlier, these perceptions suggest the need for stronger training, mentoring, and supportive supervision for healthcare providers. Improving diagnostic awareness and accountability within TB services will be essential for reducing delays and strengthening trust in care.

## Conclusion

Our research highlights the extreme challenges faced by refugee populations in Taraba State in recognizing TB symptoms, obtaining a diagnosis, and sustaining engagement with TB care. Participants described long, expensive, and often arduous pathways between symptom onset and diagnosis, frequently seeking help from multiple providers before receiving appropriate testing and treatment. Individuals demonstrate extreme tenacity in efforts to regain their health. For many participants, TB diagnosis was ultimately achieved through persistence in navigating multiple unsuccessful, costly, and sometimes dangerous care encounters. This underscores the need for widespread TB awareness campaigns in refugee communities, and for all providers, formal and informal, to be brought on board as partners in identifying people with TB symptoms and supporting access to care. Community-based TB champions and mobile services active in communities could provide further important support.

The barriers identified in this study also highlight the multiplicity of intersecting challenges refugees face in remaining in care, especially for women and older people. Individual-level pressures (hunger, transport costs, and healthcare fees) mean that continuing to attend appointments can come at the cost of meeting basic household needs, while interpersonal factors such as anticipated stigma, limited access to appropriate clothing, and language barriers make sustained engagement with providers harder to maintain over time. These pressures are compounded by health system weaknesses, including difficult geographic access, poor facility infrastructure, unreliable medication supply, and stigmatizing or under-resourced care encounters, all of which can erode trust and discourage people from returning for ongoing care. Addressing these barriers requires more than expanding diagnostic and treatment services alone: It calls for structural interventions such as decentralized or mobile care, expanded psychosocial support, and investment in basic facility infrastructure, alongside training and accountability measures that help healthcare workers treat care as a relational act—recognizing that stigmatizing a person in need of care can be as detrimental as withholding medication itself. Ultimately, our study demonstrates that treatment interruption should not be interpreted primarily as a lack of motivation or commitment among people affected by TB, but rather as the cumulative effect of structural barriers that make consistent engagement with services difficult to sustain. This recognition provides direction to changes needed.

## Data Availability

The raw data supporting the conclusions of this article will be made available by the authors, without undue reservation.

## Footnotes

1 While formal, registered, pharmacists are also accessed for care in Nigeria, we have—in line with local norms—interpreted “community pharmacists” as PMVs especially as formal pharmacies were not present in the study areas.

2 The Naira has been subject to substantial currency fluctuations. These dollar estimations are based on the currency exchange in mid-2022, when it was approximately USD 12 to N5000.
